# Long Non-Coding RNA: A Potential Strategy for the Diagnosis and Treatment of Colorectal Cancer

**DOI:** 10.3389/fonc.2021.762752

**Published:** 2021-10-27

**Authors:** Shanshan Chen, Yi Fang, Lingyu Sun, Ruonan He, Beihui He, Shuo Zhang

**Affiliations:** ^1^ The First Affiliated Hospital, Zhejiang Chinese Medical University, Hangzhou, China; ^2^ The First Clinical Medical College, Zhejiang Chinese Medical University, Hangzhou, China

**Keywords:** long non-coding RNA, colorectal cancer, drug resistance, proliferation, metastasis, occurrence, invasion

## Abstract

Colorectal cancer (CRC), being one of the most commonly diagnosed cancers worldwide, endangers human health. Because the pathological mechanism of CRC is not fully understood, there are many challenges in the prevention, diagnosis, and treatment of this disease. Long non-coding RNAs (lncRNAs) have recently drawn great attention for their potential roles in the different stages of CRC formation, invasion, and progression, including regulation of molecular signaling pathways, apoptosis, autophagy, angiogenesis, tumor metabolism, immunological responses, cell cycle, and epithelial-mesenchymal transition (EMT). This review aims to discuss the potential mechanisms of several oncogenic lncRNAs, as well as several suppressor lncRNAs, in CRC occurrence and development to aid in the discovery of new methods for CRC diagnosis, treatment, and prognosis assessment.

## Introduction

Colorectal cancer (CRC) is one of the most commonly diagnosed cancers worldwide. In the USA, CRC is the third leading cause of cancer mortality in both men and women. There will be over 10,000 new cases in 2020, and the proportion of young patients is increasing (1). In addition, the incidence rate of CRC in transitioned countries is approximately 4-fold higher than that in transitioning countries (2). Since the pathological mechanism of CRC is not yet fully understood, further studies are urgently needed to identify and develop new effective biomarkers and targets for its diagnosis and treatment.

Long non-coding RNAs (lncRNAs) are non-coding transcripts composed of more than 200 nucleotides that have a variety of regulatory modes. lncRNAs interact with proteins, RNA, and DNA and form RNA-RNA, RNA-DNA, and RNA-protein complexes, allowing them to participate in many important biological processes, such as transcription, intranuclear transport, and genomic imprinting (3, 4). Recent studies have shown that lncRNAs are involved in the regulation of CRC occurrence and development. Some oncogenic lncRNAs promote the occurrence, proliferation, invasion, metastasis, and drug resistance of CRC cells, while some lncRNAs suppress the proliferation and metastasis of CRC cells. The potential mechanism of lncRNAs in CRC are combining with proteins to form complexes to regulate the target downstream, affecting miRNA translation, acting as miRNA sponges (ceRNA) or scaffolds, regulating relevant signalling pathways and cell cycle progression, as well as the expression of transcriptional factors, ribosomal biogenesis factors, and anti-oncogenes.

Understanding the potential roles of lncRNAs in CRC occurrence, proliferation, invasion, metastasis, and drug resistance, as well as the effects of some suppressor lncRNAs related to CRC, can provide new ideas and countermeasures for the diagnosis, assessment, and treatment of CRC.

## LncRNAs in Colorectal Cancer

LncRNAs can regulate the occurrence and development of CRC. Some lncRNAs tend to promote cancer cell proliferation, invasion, metastasis, and drug resistance, while others suppress cancer cell proliferation and metastasis. We discuss their roles and related molecular mechanisms in the following sections.

### LncRNAs in Colorectal Cancer Occurrence

LncRNA ubiquitin-like plant homeodomain (PHD) and really interesting new gene (RING) finger domain-containing protein 1 (UHRF1) Protein Associated Transcript (UPAT) expression is significantly upregulated in highly tumorigenic CRC cell lines compared to that in weak tumorigenic and normal cell lines, as evaluated through quantitative reverse transcription-polymerase chain reaction (qRT-PCR) analysis. UPAT stabilizes the UHRF1 protein by interfering with the ubiquitination and degradation mediated by β-TrCP E3 ubiquitin ligase (β-TrCP1 and β-TrCP2), thus promoting the expression of stearoyl-coenzyme A desaturase-1 (SCD1) and sprouty RTK signaling antagonist 4 (SPRY4). UPAT can also upregulate the expression of phosphoglucomutase 1 (PGM1) and G protein-coupled receptor class C group 5 member A (GPRC5A), but the specific mechanism is not yet clear (5). The epigenetic factor UHRF1 regulates transcription by regulating DNA methylation and histone modification and plays a key role in tumor cell proliferation and survival (6). SCD1, SPRY4, PGM1, and GPRC5A are necessary for the transformation of normal cells into cancer cells and their survival and development (7–9). The UHRF1-UPAT axis may be a promising molecular target for the treatment of CRC. However, the regulatory mechanism of UPAT on the expression of PGM1 and GPRC5A requires further study.

Polycomb repressive complex 2 (PRC2) and DEAD box protein 5 (DDX5) associated lncRNA (PRADX), which acts as a cancer driver, is highly expressed in CRC cells and tissues and is mainly distributed in the nucleus. Enhancer of zeste homolog 2 (EZH2), a histone methyltransferase that catalyzes histone H3 lysine 27 trimethylation (H3K27me3) and epigenetically silences target genes (10), is overexpressed in many cancer types and has been shown to act as an oncogene (11, 12). PRADX can bind to the EZH2 protein through its 1-500 bp 5’ end sequence to recruit PRC2 and DDX5, forming a PRC2/DDX5 complex (13). Activation of the nuclear factor kappa B (NF-κB) pathway promotes the occurrence of colorectal adenocarcinoma. UBXN1 is a UBX domain protein that can inhibit the degradation of IκBα thus blocking the NF-κB pathway (14). Because the PRC2/DDX5 complex can inhibit the expression of UBXN1, the NF-κB pathway is activated thereby promoting the occurrence of colon adenocarcinoma (15).

LncRNA antisense ncRNA in the abundant in neuroepithelium area (ANA)/B-cell translocation gene 3 (BTG3) locus (ASBEL) is a tumorigenic gene that can be directly activated by the Wnt/β-catenin pathway. At the same time, β-catenin, can also activate the transcription factor TCF3 (16). TCF3 can form a complex with ASBEL to downregulate the expression of the target transcription factor ATF3 and inhibit the development of CRC. The classic Wnt/β-catenin signaling pathway plays an important role in regulating proliferation, cell fate, stem and progenitor cell self-renewal, and tumorigenesis (17–19). The role of this signaling pathway in carcinogenesis was first described in the context of adenomatous polyposis coli (APC) gene mutations. APC mutations are usually acquired early in the onset of most colon cancers (over 80%), leading to the cytoplasmic accumulation of β-catenin, which binds to TCF/Lef1 and shuttles to the nucleus, where it acts as a transcription factor and promotes cell proliferation (20). Thus, the β-catenin-ASBEL-TCF3-ATF3 pathway may be a promising target for colon cancer treatment.

Cancer susceptibility 21 (CASC21) is significantly upregulated in CRC tissues. Yes1 Associated Transcriptional Regulator (YAP1), a well-studied transcriptional coactivator, is a main downstream effector of the Hippo pathway that plays a critical role in controlling organ size in animals (21). YAP1 is known to be upregulated in some solid tumors, including CRC, and acts as an oncogene that promotes tumor cell proliferation, migration, and invasion (22, 23). Therefore, the upregulation of YAP1 promotes the occurrence of CRC. lncRNAs can regulate the mRNA expression of many functional target genes by functioning as competing endogenous RNAs (ceRNAs) that sponge microRNAs (miRNAs) and competitively inhibit the binding of miRNAs to targets (24, 25). miRNAs are short non-coding RNA molecules that inhibit the expression of target genes by cutting down mRNA or inhibiting translation (26). CASC21 acts as a ceRNA sponging miR-7-5p to upregulate YAP1 expression and thus promote the occurrence of CRC (27).

**Figure 1:**
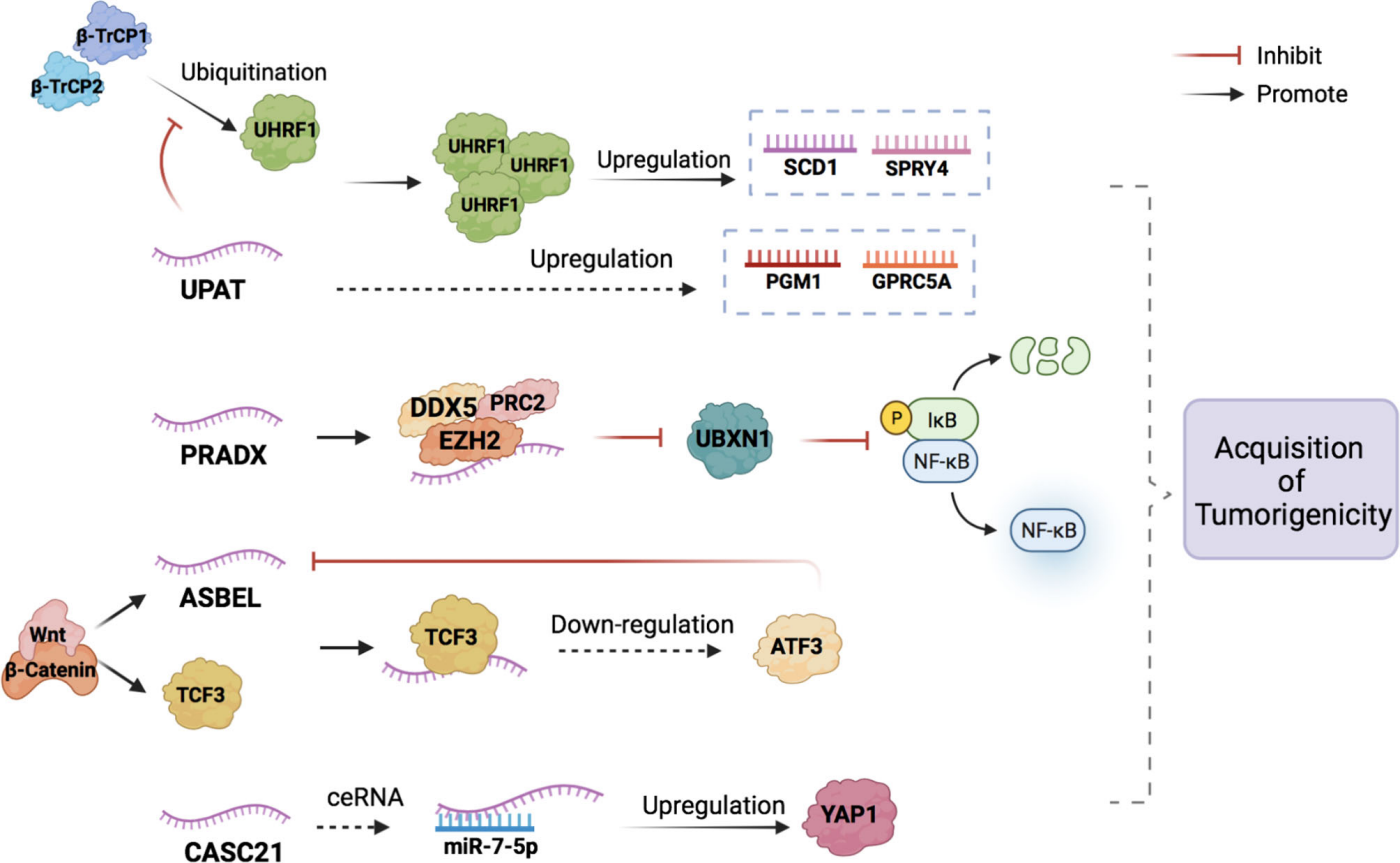
The potential mechanisms of lncRNAs in the CRC occurrence. LncRNAs participate in the acquisition of tumorigenicity of CRC by combining with proteins to form complexes and acting as miRNA sponges (ceRNA).

The finding of lncRNA tumorigenicity helps us detect CRC at an early stage, and related molecular signaling pathways may provide us with potential targets for treatment (**Figure 1**, **Table 1**).

**Table 1:** LncRNAs in the occurrence, proliferation, invasion, and metastasis of CRC cells.

lncRNAs	Expression	Function	Downstream targets	Reference
UPAT	↑	Promotes tumorigenesis	SCD1、SPRY4 PGM1、GPRC5A	(5)
PRADX	↑	Promotes tumorigenesis	EZH2	(6)
ASBEL	↑	Promotes tumorigenesis	ATF3	(15)
CASC21	↑	Promotes tumorigenesis	mir-7-5p/Yap1	(14)
GLCC1	↑	Promote glucose metabolism, proliferation	c-Myc	(16)
XIST	↑	Promote proliferation, EMT	mir-486-5p	(28)
CRNDE	↑	Promote proliferation, Drug resistance	mir-181a-5p	(17)
SNHG3	↑	Promote proliferation, metastasis	mir-539/Runx2	(18)
BANCR	↑	Promote proliferation Drug resistance	mir-203/CSE1L	(19)
CCAT2	↑	Promote proliferation, drug resistance	mir-145, BOP1 MYC	(20, 29)
P14AS	↑	Promote proliferation	lncRNA ANRIL	(22)
00659	↑	Promote proliferation	/	(30)
LOC441461	↑	Promote proliferation, metastasis	RhoA/ROCK MLC,LIMK1	(31)
PRNCR1	↑	Promote proliferation, Regulates cell cycle	/	(32)
SNHG1	↑	Promote proliferation	mir-154-5p/EZH2	(33)
CYTOR	↑	Increases migration, invasion	Wnt/β-Catenin	(34)
RAMS11	↑	Promote metastasis	TOP2α	(35)
LDLRAD4-AS1	↑	Promote metastasis, EMT	snail, E-cadherins	(36)
CALIC	↑	Promote metastasis	AXL	(37)
GSEC	↑	Promote metastasis	DHX36	(38)
ZEB1-AS1	↑	Enhances growth, metastasis	mir-455-3p/PAK2	(39)
LINCO1578	↑	Promote metastasis	NF-κB/YY1	(40)

### LncRNAs in Colorectal Cancer Cell Proliferation

Glycolysis-associated lncRNA of colorectal cancer (GLCC1) is an oncogene in CRC, which is involved in the glycolysis of CRC cells (30). Abnormal activation of glycolytic pathways in cancer cells is considered a sign of malignancy (31). GLCC1 stabilizes c-Myc by binding to HSP90 (HSP90AA1) chaperone and preventing cytoplasmic ubiquitination degradation, thereby increasing the transcription level of lactate dehydrogenase A (LDHA) and activating glycolytic metabolism. HSP90, as a protein chaperone, can stabilize transcription factors, protein kinases, and oncoproteins in the tumor signaling pathway (32). C-myc is an important oncogene involved in regulating glucose metabolism and is the key on/off switch in cancer cell metabolism. High levels of LDHA expression are associated with poor clinical outcomes of CRC due to its regulation of glycolytic metabolism in cancer cells (41, 42). GLCC1-c-Myc-LDHA, as a cascade reaction coordinated by GLCC1 under glucose starvation, may be a promising metabolic blocker target for antitumor therapy.

LncRNA X-inactive specific transcript (XIST) expression in CRC tissues is abnormally high. XIST can target and downregulate mir-486-5p, leading to CRC cell proliferation (43). The miRNA miR-486-5p plays a protective role against CRC, and it acts by obstructing the expression of neuropilin‐2 (NRP-2) (44), which is known to interfere with the epithelial-mesenchymal transition (EMT) of CRC cells *via* crosstalk with transforming growth factor β1 (TGF-β1) (33).

The lncRNA colorectal differentially expressed (CRNDE) was originally found to be highly expressed in colorectal adenomas and adenocarcinomas (34). CRNDE targets and upregulates miR-181a-5p. Overexpression of miR-181a-5p reduces the expression of endogenous β-catenin and TCF4, leading to the inhibition of the Wnt/β-catenin signaling pathway. CRNDE binds to miR-181a-5p and blocks its inhibitory effect on Wnt/β-catenin signaling, resulting in the proliferation of CRC cells (35). From this perspective, we can hypothesize that it is possible to search for miRNA agonists that can compete against miR-181a-5p in binding with CRNDE to inhibit the Wnt/β-catenin signaling pathway on the proliferation of colon cancer cells.

LncRNA small nuclear RNA host gene 3 (SNHG3) expression in CRC tissue has been found to be significantly upregulated compared to that in adjacent normal tissues. The binding of SNHG3 and miR-539 can upregulate the expression of its target gene, runt-related transcription factor 2 (RUNX2), to promote cancer cell proliferation (45). RUNX2 is involved in the occurrence and development of a variety of cancers, including CRC, and plays a role as an oncogene (36, 37). The authors believe that interfering with the ceRNA mechanism of SNHG3 and blocking its inhibitory effect on miRNA may help suppress the development of CRC.

The expression of lncRNAs BRAF-activated non-coding RNA (BANCR) and chromosome segregation like 1 (CSE1L) has also been found to be significantly upregulated in CRC tissues. BANCR acts as a molecular sponge for miR-203 and separates miR-203 from CSE1L in CRC cells, thus upregulating the expression of CSE1L (46). The upregulation of CSE1L expression, in turn, can promote the proliferation and invasion of cancer cells (38, 47). BANCR knockdown suppressed CRC cell proliferation, and CSE1L overexpression reversed the anti-proliferation and anti-invasion effects of BANCR silencing (46). Thus, the upregulation of BANCR may promote CRC development through the miR-203/CSE1L axis.

The lncRNA colon cancer-associated transcript 2 (CCAT2), which is overexpressed in CRC tissues, can promote cancer cell proliferation (39). Yu et al. found that miR-145 could modulate the proliferation and differentiation of colon cancer stem cells (CSCs). CCAT2 can selectively inhibit miR-145 maturation by preventing the export of pre-miR-145 to the cytoplasm (39).

P14AS, which binds to AU-rich binding factor 1 (AUF1), is a novel lncRNA transcribed from the antisense strand of the CDKN2A/P14 gene (48). AUF1 is an RNA-binding protein that promotes the expression of many cancer-related RNAs, including c-Myc, P16, and NEAT1 (40, 49, 50). The carcinogenicity of lncRNA ANRIL (CDKN2B-AS1) has been confirmed in various studies (51, 52), and the binding of lncRNA P14AS and AUF1 can increase the levels of ANRIL, resulting in cancer cell proliferation.

Recently, many studies have found that the knockdown of some lncRNAs, such as lncRNA 00659, LOC441461, and lncRNA prostate cancer−associated non−coding RNA 1 (PRNCR1), can inhibit cancer cell proliferation by interfering with the process of the cell cycle (53–55). Among these, the knockdown of LOC441461 expression inhibits the phosphorylation of MLC and LIMK1 by inhibiting RhoA/ROCK signaling, thereby affecting the cell cycle and inducing apoptosis to prevent the proliferation of cancer cells (54).

SNHG1 expression was found to be significantly upregulated in CRC tissues while Kruppel-like factor 2 (KLF2) was downregulated. KLF2 possesses tumor-suppressor features, such as inhibition of cell proliferation and enhancement of DNA damage-associated apoptosis in many cancers (56). SNHG1 can directly interact with EZH2 to silence KLF2 expression and promote CRC proliferation (29).

**Figure 2:**
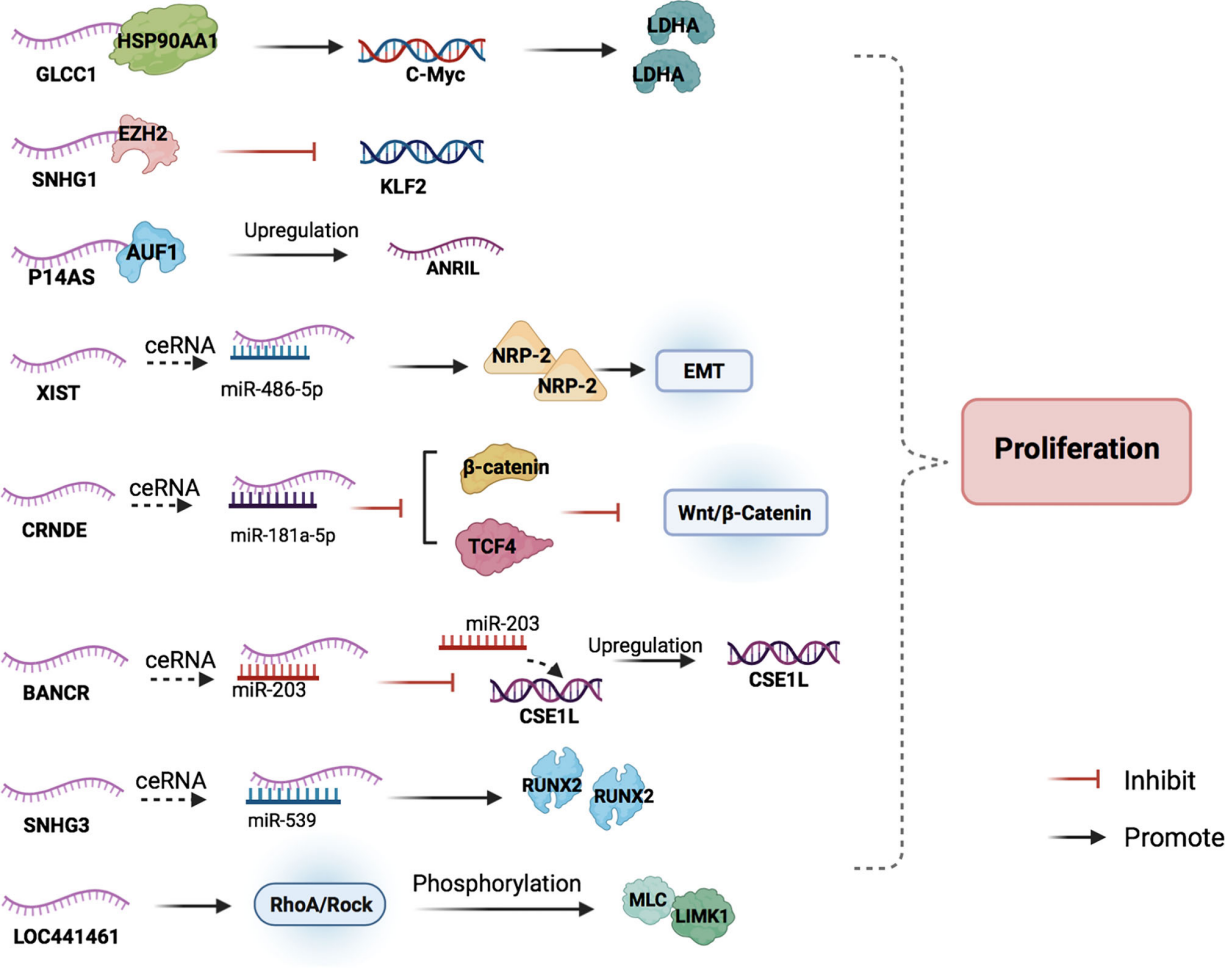
The main mechanisms of lncRNAs in CRC cell proliferation. LncRNAs participate in the CRC cell proliferation by combining with proteins to regulate the target downstream, acting as miRNA sponges, and blocking cell cycle progression.

The discovery that lncRNAs promote CRC cell proliferation may help improve the prognosis of patients. Studying their specific mechanisms may contribute to the development of new therapeutic targets to inhibit the proliferation of tumor cells (**Figure 2**, **Table 1**).

### LncRNAs in Colorectal Cancer Cell Invasion and Metastasis

The lncRNA cytoskeleton regulator RNA (CYTOR) is involved in CRC cell invasion and metastasis *via* the Wnt/β-catenin pathway. CYTOR binds to cytoplasmic β-catenin and inhibits its phosphorylation induced by kinase 1 (CK1), resulting in β-catenin accumulation and translocation to the nucleus. At the same time, β-catenin enhances CYTOR transcriptional activity in the nucleus, thus forming a positive feedback loop. The wound-healing assay showed that CYTOR knockdown inhibited the migration of HCT8 and SW620 cells, and the depletion of CYTOR colon cancer cells failed to induce further invasion. The nude mouse model for lung metastases inoculated with CYTOR-expressing cancer cells showed that the CYTOR knockdown group had a decrease in the size and number of metastatic tumor nodules compared with the control group. This illustrated that CYTOR promoted CRC cell invasion and metastasis both *in vivo* and *in vitro*. The upregulation of CYTOR accelerates β-catenin nuclear translocation and increases the transcription activity of the β-catenin/TCF complex in the nucleus, activating the Wnt/β-catenin pathway to promote cancer cell invasion and metastasis (57).

RNA associated with metastasis 11 (RAMS11) is a lncRNA that can induce tumor formation and promote tumor growth and metastasis. Compared to that in primary tumor tissues, the expression level of RAMS11 was increased in metastatic colon cancer cells. Upon measuring samples from two independent cohorts of colon cancer patients through qPCR, the expression of RAMS11 in metastatic samples was upregulated compared with that in primary cancer tissues. RAMS11 promotes topoisomerase II alpha (TOP2α) expression by binding to Chromobox protein 4 (CBX4). Silencing CBX4 or TOP2α can slow down the invasion and metastasis of LoVo colon cancer cell lines (58). TOP2α is used as a proliferation marker for many cancer types, including CRC (59, 60), and its increased expression levels are associated with prostate cancer, pancreatic cancer, and breast cancer metastases (61–64). In patients with primary and metastatic CRC (mCRC), the expression of TOP2α is elevated (65–67). RAMS11 promotes the resistance of colon cancer cells to topoisomerase inhibitors, which has become the basic principle for the use of anthracyclines to treat certain mCRC patients (58).

LDLRAD4 antisense RNA 1 (LDLRAD4-AS1) expression levels were reported to be higher in rectal cancer tissues than in adjacent normal tissues. Using the Transwell assay, Mo et al. found that the overexpression of the lncRNA LDLRAD4-AS1 promoted the migration and invasion of highly invasive CRC cell lines (RKO and LoVo) *in vitro*. LDLRAD4-AS1 also enhanced the migration ability of RKO and LoVo cells in a wound-healing assay. The upregulation of lncRNA LDLRAD4-AS1 expression destabilizes LDLRAD4 mRNA and decreases LDLRAD4 mRNA expression at the protein level, thus reducing the transcription factor snail and E-cadherins, which then promotes epithelial interstitialization and metastasis (68). EMT, marked by the loss of E-cadherin, enables the epithelial cells of a primary tumor to lose cell polarity and break the cellular adhesion constraints, allowing cancer cells to acquire migratory and invasive characteristics and be mesenchymal-like towards aggressive malignancy (69–71).

The lncRNA cancer metastasis‐associated long intergenic non‐coding RNA (CALIC) was significantly upregulated in subpopulations of HCT116 cells that were selected for their elevated metastatic activity. The RNA-Seq and gene ontology (GO) analysis on HCT116 cells that used small interfering RNA (siRNA) to knock down CALIC showed that CALIC target genes are enriched in genes involved in “cell movement” and “cell localization” and that CALIC knockdown inhibits high-level expression CALIC’s WiDr colon cancer cell migration. In contrast, the knockdown had little effect on the migration of Caco-2 and Caco-320 colon cancer cells that expressed low levels of CALIC (72). The receptor tyrosine kinase AXL, which regulates FAK1, RHO family GTPases, and GTP exchange factor Vav1, is important in cancer cell migration and invasion. CALIC associates with the RNA‐binding protein heterogeneous nuclear ribonucleoprotein L (hnRNP‐L) and upregulates AXL, thereby promoting migration and metastasis of colon cancer cells (73, 74).

G-quadruplex forming sequence containing lncRNA (GSEC) is enriched in the cytoplasm of colon cancer cells, and its expression is significantly higher than that in the surrounding normal colon tissue. DHX36 is a GSEC-related protein that has the ability to unfold G-quadruplex. Overexpression of DHX36 inhibits the motility of the colon cancer cell line DLD-1. GSEC is a G-quadruplex-containing lncRNA that can bind to DHX36, thus inhibiting the unwinding activity of G-quadruplexes to promote colon cancer cell metastasis (28).

The knockdown of the lncRNA zinc finger E-box binding homeobox 1 antisense 1 (ZEB1-AS1), whose expression level increased in colon adenocarcinoma tissues, suppressed the invasion and migration of SW480 and HT29 cells (75). P21-activated kinase 2 (PAK2), a member of the P21-activated kinase (PAK) family of serine/threonine kinases, engages in many signaling pathways related to malignant progression. A study showed that miR-455-3p exhibits anti-cancer effects in colon adenocarcinoma cells by targeting PAK2. ZEB1-AS1 inhibits miR-455-3p and dampens the inhibition of PAK2, thereby promoting cancer cell metastasis (76).

LncRNA LINC01578 activates NF-κB, which, in turn, promotes the expression of LINC01578. Therefore, a positive feedback loop forms between LINC01578 and NF-κB/YY1 (77). NF-κB has been shown to promote colon cancer cell invasion and metastasis (78, 79), and LINC01578 also exerts a similar effect due to the feedback loop (77).

**Figure 3:**
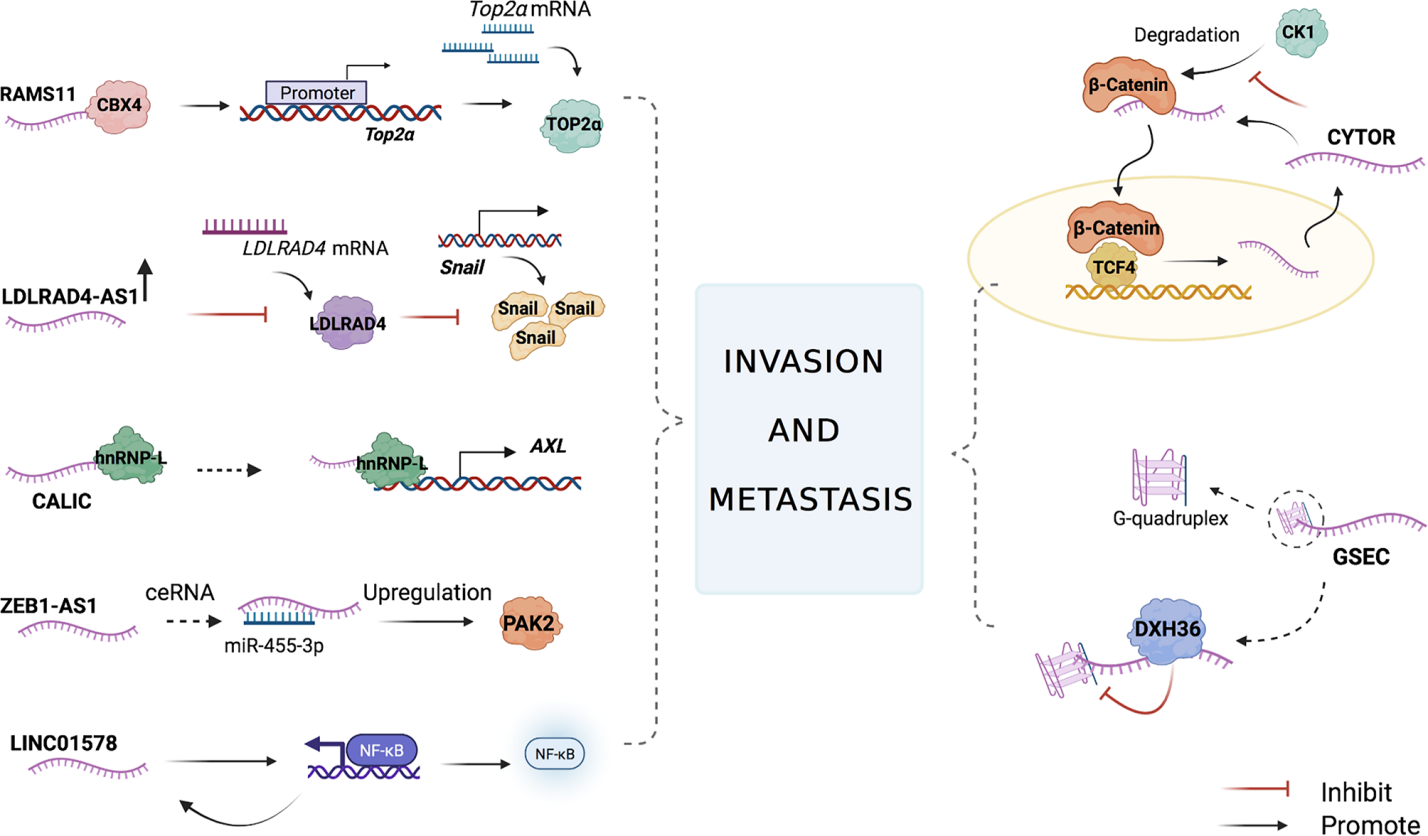
The main mechanisms of lncRNAs in CRC cell invasion and metastasis. LncRNAs participate in the CRC cell invasion and metastasis by combining with proteins to regulate the target downstream, affecting miRNA translation, acting as miRNA sponges, regulating the expression of transcriptional factors, and activating nuclear factor kappa-B (NF-kB).

Studying the signaling pathways of lncRNAs in promoting CRC cell metastasis may help us find potential therapeutic targets for treatment and predict the metastasis rate of the disease (**Figure 3** and **Table 1**).

### LncRNAs in Colorectal Cancer Cell Drug Resistance

Currently, improvements in screening, surgical techniques, radiation therapy, and chemotherapy largely contribute to the reduction in the mortality rate of colorectal cancer; however, over 40% of colorectal cancer patients die from recurrence and metastasis, and multiple-drug resistance in chemotherapy is the main reason for treatment failure. There is strong evidence that supports the effect of lncRNA on the increase in drug resistance in CRC cells (80) (**Table 2**).

**Table 2:** LncRNAs in CRC cell drug resistance.

lncRNAs	Expression	Function	Downstream targets	Reference
CCAT2	↑	Promotedrug resistance, proliferation	mir-145, BOP1 MYC	(20, 29)
H19	↑	Promotes drug resistance	mir-141	(52)
MALAT1	↑	Promotes drug resistance	mir-128	(54)
Linc00152	↑	Promotes drug resistance	mir-193a-3p, ERBB4	(57)
MIR4435	↑	Promotes drug resistance	Nrf2/HO-1	(58)
POU6F2AS	↑	Promote proliferation, drug resistance	P-gp, MRP2, BRCA2	(62)

LncRNA H19 is overexpressed in CRC cells and is associated with the immunostaining score of acetaldehyde dehydrogenase 1A1 (ALDH1A1) in H19-high and H19-low CRC specimens (81). ALDH1A1 is a cancer stem cell marker in colon cancer cells (82), which suggests that H19 is also associated with the malignant potential of CRC stem cells. Studies have evaluated the chemosensitivity of CRC cells in the routine and widespread use of oxaliplatin, and it has been found that H19 overexpression promotes oxaliplatin resistance in SW480 and HCT116 cells. In addition, an apoptosis assay confirmed that H19 overexpression enhanced oxaliplatin resistance in SW480 cells. Cancer-associated fibroblasts (CAFs) are the main type of stromal cells in the CRC tissue matrix, which can transfer H19 into cancer cells by secreting H19-containing exosomes. H19 can then activate the β-catenin pathway by acting as a ceRNA to sponge miR-141, thus increasing drug resistance in cancer cells (81).

The lncRNA metastasis-associated lung adenocarcinoma transcript 1 (MALAT1) is associated with a poor response to oxaliplatin-based chemotherapy in CRC patients. A study measured the expression level of MALAT1 in the serum of 53 patients with metastatic CRC using qRT-PCR. According to the Response Evaluation Criteria in Solid Tumors (RECIST) criteria, patients were divided into two groups: responsive (CR + PR) and non-responsive (SD + PD) groups. The results showed that the non-responsive group had significantly higher MALAT1 expression levels compared with the responsive group (83). During oxaliplatin treatment, MALAT1 binds EZH2 to the CDH1 promoter and inhibits miR-128 to promote drug resistance in colorectal cancer cells (84).

The lncRNA CCAT2 promotes the expression of genes involved in ribosome biogenesis and protein synthesis. A CRC cohort study found that lncRNA CCAT2 was positively associated with the expression of BOP1 ribosomal biogenesis factor (BOP1). Chromosomal instability (CIN) can increase chemotherapy sensitivity in colon cancer cells (85). BOP1 can increase the active form of aurora B kinase, which is responsible for regulating chromosomal segregation and promoting CIN, thus increasing drug resistance (86). lncRNA CCAT2 directly interacts and stabilizes BOP1. In addition, it activates the expression of BOP1 by increasing MYC expression.

Lnc00152 is highly expressed in SW620 and HT29 cells at the basal level compared to SW480 and Caco2 cells. As for apoptosis-related genes, Western blot analysis of cleaved poly (ADP-ribose) polymerase (PARP) and cleaved Caspase 3 revealed that SW480 and Caco2 cells are more sensitive to oxaliplatin-induced apoptosis than SW620 and HT29 cells. It has been shown that Lnc00152 upregulates ERBB4 by competitively binding to miR-193a-3p and then activates the AKT pathway, thereby leading to the development of resistance to oxaliplatin (87).

LncRNA MIR4435 expression in cisplatin-resistant colon cancer HCT116 cells is seven to eight times higher than that in normal colon cancer cells, as determined through PCR analysis (88). Nuclear factor erythroid 2-related factor 2 (Nrf2) is a transcription factor that responds to oxidative stress and plays a crucial role in redox homeostasis (89). Heme oxygenase (HO-1), which is downstream of Nrf2, also plays a role (90). Both are associated with drug resistance and poor prognosis. The lncRNA MIR4435 may increase the cisplatin resistance of colon cancer cells by promoting the expression of Nrf2/HO-1 (91).

LncRNA POU6F2-antisense 2 (POU6F2-AS2) knockdown in colon cancer cell lines leads to the downregulation of the expression of the anti-cancer genes P-gp, MRP2, and BRCA2. Hence, knockdown of lncRNA POU6F2-AS increases the sensitivity of colon cancer cells to cisplatin (92).

**Figure 4:**
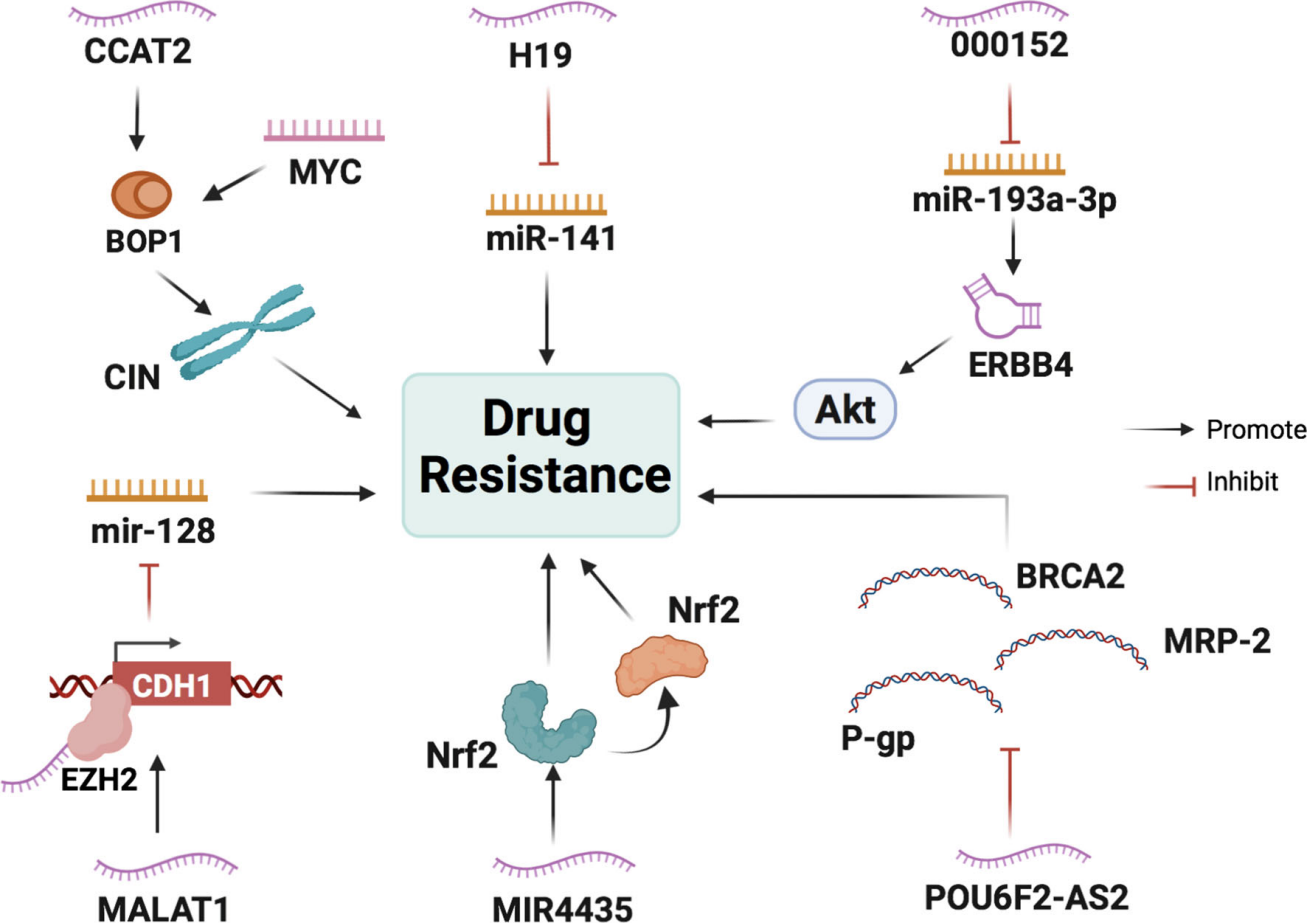
The main mechanisms of lncRNAs in CRC cell drug resistance. LncRNAs participate in CRC cell drug resistance by acting as scaffolds or miRNA sponges and regulating the expression of transcriptional factors, ribosomal biogenesis factors, and anti-oncogenes.

Studies on lncRNAs’ regulation of chemoresistance in CRC can help predict drug sensitivity to different chemotherapy treatments in patients with CRC and can serve as a guide in adjusting drug use, modifying treatments, and ultimately improving chemotherapy (**Figure 4**, **Table 2**).

### Inhibitory lncRNAs in Colorectal Cancer

LncRNAs function not only as oncogenes but also as tumor suppressor genes. Promoting the expression of tumor suppressor lncRNAs may provide a new direction for the treatment of CRC.

LncRNA P53 induced transcript (PINT) expression in primary CRC cells is downregulated compared to that in normal colorectal tissues. The lncRNA PINT is the target of the transcription factor P53, which plays a crucial role in cancer suppression. An *in vitro* study showed that colon cancer HCT0116 cells overexpressing PINT had a slower growth rate than control cells, but the underlying anti-cancer mechanism has not been revealed (93).

LncRNA loc285194, which is significantly downregulated in CRC cells compared with normal tissues, can also be induced by transcription factor P53. Overexpression of loc285194 inhibited colon cancer HCT-116 and MCF-7 cell proliferation by negatively regulating mir-211 (94).

Maternally expressed 3 (MEG3) is a lncRNA that enhances the sensitivity of CRC cells to chemotherapeutic agents. Its expression in CRC tissues is increased compared with that in adjacent normal tissues. MEG3 knockdown promotes the proliferation, migration, and colony formation of CRC cells and induces G0/G1 cell cycle arrest in these cells (95). MEG3 also acts as a sponge miR-141 to increase the expression of PDCD4 in CRC, which enhances the sensitivity of CRC HCT116 and HT29 cells to oxaliplatin (96).

LncRNA overexpressed in colon carcinoma-1 (OCC-1) knockdown in CRC cells promotes the growth of cancer cells (97). Human antigen R (HuR) is an RNA-binding protein that can stabilize mRNAs involved in a variety of biological processes. OCC-1 binds to the HuR protein and increases its interaction with E3 ubiquitin ligase β-TrCP1, leading to the ubiquitination and degradation of HuR protein, which suppresses the progression of CRC (98).

The lncRNA tumor suppressor candidate 7 (TUSC7) is involved in inhibiting the migration and invasion of CRC cells. EMT is a key process in inducing migration and invasion (99, 100). Zhang et al. detected EMT biomarkers through qRT-PCR and Western blot analysis in CRC cells and discovered that the expression level of TUSC7 is positively associated with the expression of E-cadherin and negatively associated with the expression of vimentin. This indicates that TUSC7 can inhibit EMT in CRC cells to suppress invasion and metastasis (101).

The lncRNA DPP10 antisense RNA 1 (DPP10-AS1) exerts anti-tumor effects on colon cancer cells. It can downregulate the expression of its target gene, miR-127-3p, to increase adenylate cyclase 1 (ADCY1). Thus, it can suppress the proliferation, migration, and invasion of CSCs and increase apoptosis (102).

**Figure 5:**
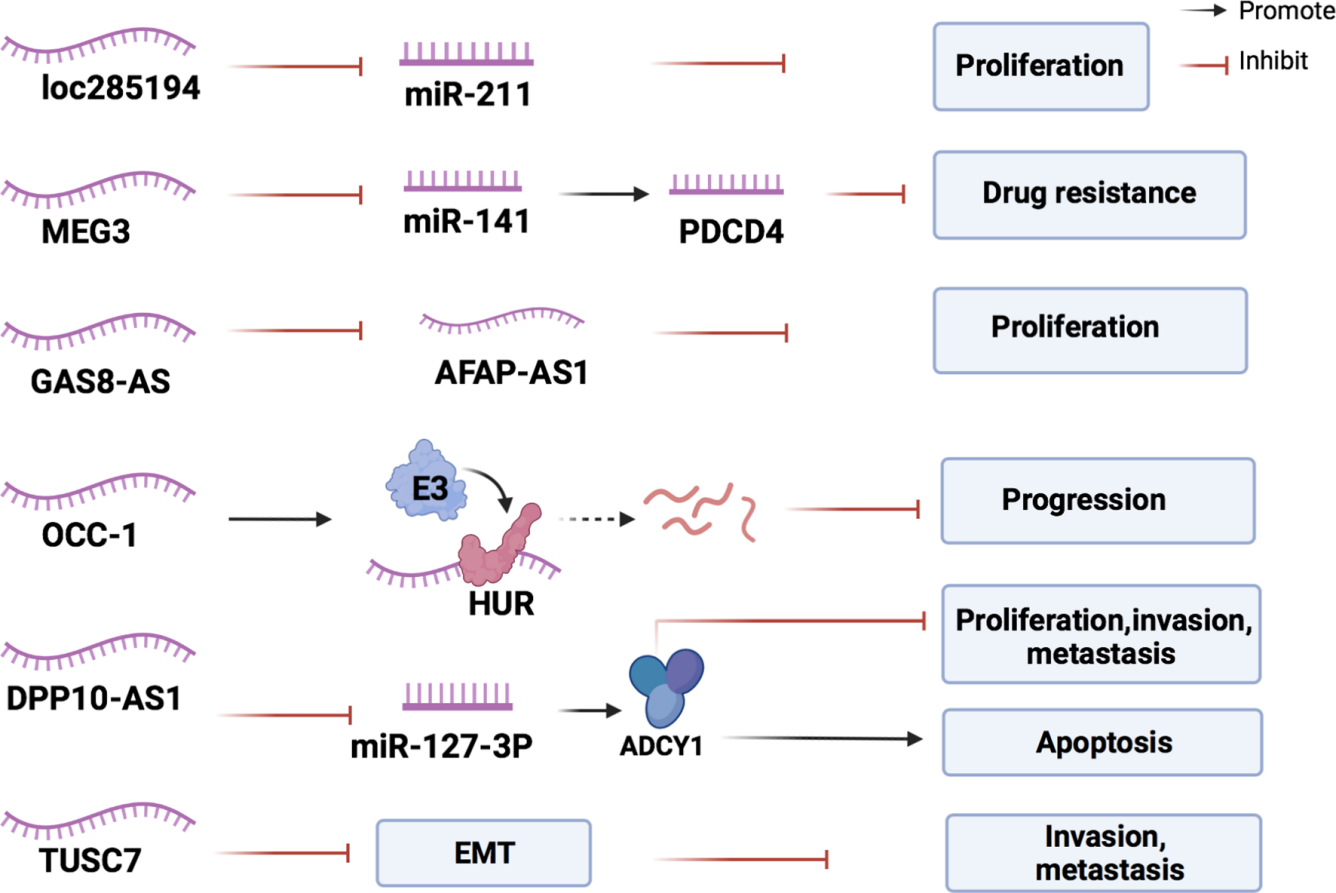
The mechanisms of lncRNAs in inhibiting the development of CRC. Inhibitory lncRNAs influence the expression of miRNAs, lncRNAs, and proteins associated with CRC proliferation, and inhibit the intraepithelial mesenchymal transformation of CRC cells to resist cancer progression.

By identifying more inhibitory lncRNAs in CRC, we can explore the possibility of inhibiting tumor progression by upregulating their expression (**Figure 5** and **Table 3**).

**Table 3:** Inhibitory lncRNAs in CRC.

lncRNAs	Expression	Function	Downstream targets	Reference
Pint	↓	Inhibits proliferation	/	(63)
Loc285194	↓	Inhibits proliferation	mir-211	(64)
MEG3	↓	Inhibits proliferation, metastasis, drug resistance	mir-141/PDCD4	(65, 66)
OCC-1	↓	suppresses growth	HUR	(69)
TUSC7	↓	Inhibits metastasis	EMT	(73)
DPP10-AS1	↓	Inhibits metastasis, proliferation	mir-127-3p	(74)

## Conclusion

Numerous studies have shown that lncRNAs play an important role in the occurrence and development of CRC. Studying the effects of lncRNAs on cancer cell proliferation, invasion, metastasis, and drug resistance, with a focus on the related signaling pathways, can help us to further understand CRC and develop better treatment strategies. For example, lncRNA HOX antisense intergenic RNA (HOTAIR) is involved in cancer cell proliferation, apoptosis, invasion, and metastasis, and its increased expression in the blood is associated with a high mortality rate (103–105). Propofol can inhibit lncRNA HOTAIR and is a potential drug for CRC treatment (106). lncRNA urothelial carcinoma associated 1 (UCA1) also plays a critical role in tumorigenesis (107), and it can be inhibited by metformin resulting in the apoptosis of CRC cells (108). Curcumin, which exists in the rhizomes of *Curcuma longa*, has been approved for the treatment of CRC (109, 110), and it can inhibit tumor occurrence by inducing the expression of a suppressor lncRNA neighbor of BRCA1 gene 2 (NBR2).

Recent studies have reported that lncRNAs can be encapsulated in exosomes and transmitted among tumor cells, regulating the occurrence and development of tumors. Exosomal lncRNAs show high organ specificity in the blood, urine, saliva, and tumor tissue and have the advantages of being non-invasive, repeatably detectable, and real-time monitoring. Thus, exosomal lncRNAs are expected to function as meaningful biomarkers (111–113). CRNDE-h is an exosomal lncRNA that can effectively distinguish CRC patients from benign colorectal diseases and NC subjects with significantly high sensitivity and specificity. In addition, the combination of several tumor markers, such as lncRNA ZFAS1, SNHG11, LINC00909, and LINC00654, can improve the accuracy of CRC diagnosis. Moreover, increased lncRNA SNHG11 helps to further screen and diagnose CRC (109). The SLS model based on 52 lncRNAs can also predict the risk of CRC occurrence and mortality (110).

However, most studies at present can only indicate that there is a correlation between lncRNAs and CRC, but less clearly describes the specific mechanism between them. For the lncRNAs that have been studied, it is still difficult to determine which pathway is dominant, leading to increased challenges in choosing between targeted blocking or activation. A comprehensive understanding of the effects of lncRNAs on CRC has not been established, and studies on inhibitory lncRNAs are limited. It is necessary to consider the mechanism leading to the abnormal expression of lncRNAs in CRC. The upstream regulatory mechanisms of lncRNAs have not yet been fully elucidated. Therefore, new directions for future research may focus on the upstream regulatory factors of lncRNAs in CRC. These efforts may address the gaps in knowledge on lncRNA-related mechanisms and build a framework for understanding the effects of lncRNAs on CRC.

## Author Contributions

SC, BH, and SZ were involved in the conception of the study. SC, YF, LS, and RH were involved in writing the article. SC, BH, and SZ critically revised the manuscript. All authors read and approved the final manuscript. SC, YF, and LS contributed equally to this work.

## Funding

This work was supported in part by the National Natural Science Foundation of China (81973598, 82074186, 82074214). Funding was also provided by the Medicine and Health Science and Technology Plan Projects in Zhejiang province (2021KY834), and Research Fund Project of Zhejiang Chinese Medical University (2019ZY02, 2020ZG41).

## Conflict of Interest

The authors declare that the research was conducted in the absence of any commercial or financial relationships that could be construed as a potential conflict of interest.

## Publisher’s Note

All claims expressed in this article are solely those of the authors and do not necessarily represent those of their affiliated organizations, or those of the publisher, the editors and the reviewers. Any product that may be evaluated in this article, or claim that may be made by its manufacturer, is not guaranteed or endorsed by the publisher.
